# Wnt/β-catenin and NFκB signaling synergize to trigger growth factor-free regeneration of adult primary human hepatocytes

**DOI:** 10.1097/HEP.0000000000000648

**Published:** 2023-10-23

**Authors:** Nuria Oliva-Vilarnau, Christian M. Beusch, Pierre Sabatier, Eirini Sakaraki, Amelie Tjaden, Lukas Graetz, Florian A. Büttner, Debra Dorotea, My Nguyen, Filip Bergqvist, Yvonne Sundström, Susanne Müller, Roman A. Zubarev, Gunnar Schulte, Claudia Tredup, Roberto Gramignoli, Uwe J.F. Tietge, Volker M. Lauschke

**Affiliations:** 1 Department of Physiology and Pharmacology, Karolinska Institutet, Stockholm, Sweden; 2 Department of Medical Biochemistry and Biophysics, Karolinska Institutet, Stockholm, Sweden; 3 Institute of Pharmaceutical Chemistry, Johann Wolfgang Goethe University, Frankfurt am Main, Germany; 4 Buchmann Institute for Molecular Life Sciences and Structural Genomics Consortium (SGC), Frankfurt am Main, Germany; 5 Dr Margarete Fischer-Bosch Institute of Clinical Pharmacology, Stuttgart, Germany; 6 University of Tübingen, Tübingen, Germany; 7 Department of Laboratory Medicine, Division of Clinical Chemistry, Karolinska Institutet, Stockholm, Sweden; 8 Department of Medicine, Karolinska Institutet, and Karolinska University Hospital, Stockholm, Sweden; 9 The Structural Genomics Consortium (SGC), Karolinska Institutet, Stockholm, Sweden; 10 Department of Laboratory Medicine, Division of Pathology, Karolinska Institutet, Stockholm, Sweden; 11 Clinical Pathology and Cancer Diagnosis Unit, Karolinska University Hospital, Stockholm, Sweden; 12 Clinical Chemistry, Karolinska University Laboratory, Karolinska University Hospital, Stockholm, Sweden

## Abstract

**Background and Aims:**

The liver has a remarkable capacity to regenerate, which is sustained by the ability of hepatocytes to act as facultative stem cells that, while normally quiescent, re-enter the cell cycle after injury. Growth factor signaling is indispensable in rodents, whereas Wnt/β-catenin is not required for effective tissue repair. However, the molecular networks that control human liver regeneration remain unclear.

**Methods:**

Organotypic 3D spheroid cultures of primary human or murine hepatocytes were used to identify the signaling network underlying cell cycle re-entry. Furthermore, we performed chemogenomic screening of a library enriched for epigenetic regulators and modulators of immune function to determine the importance of epigenomic control for human hepatocyte regeneration.

**Results:**

Our results showed that, unlike in rodents, activation of Wnt/β-catenin signaling is the major mitogenic cue for adult primary human hepatocytes. Furthermore, we identified TGFβ inhibition and inflammatory signaling through NF-κB as essential steps for the quiescent-to-regenerative switch that allows Wnt/β-catenin-induced proliferation of human cells. In contrast, growth factors, but not Wnt/β-catenin signaling, triggered hyperplasia in murine hepatocytes. High-throughput screening in a human model confirmed the relevance of NFκB and revealed the critical roles of polycomb repressive complex 2, as well as of the bromodomain families I, II, and IV.

**Conclusions:**

This study revealed a network of NFκB, TGFβ, and Wnt/β-catenin that controls human hepatocyte regeneration in the absence of exogenous growth factors, identified novel regulators of hepatocyte proliferation, and highlighted the potential of organotypic culture systems for chemogenomic interrogation of complex physiological processes.

## Graphical abstract

**Figure:**
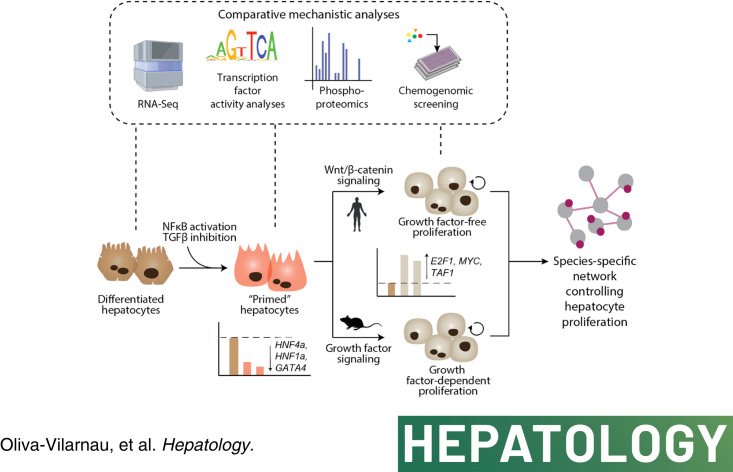

## INTRODUCTION

The liver has an outstanding ability to regenerate following trauma. The rodent partial hepatectomy model, in which 70% of the liver mass is surgically removed, is the major experimental model for studying the molecular underpinnings of liver regeneration. In this model, most of the remaining hepatocytes re-enter the cell cycle within 24 hours and undergo compensatory hyperplasia.^1,2^ This coordinated proliferation after injury is in stark contrast to homeostatic conditions under which hepatocytes are quiescent, with an average lifespan of 200–400 days.^3^ *In vivo*, hepatocytes can be regarded as facultative stem cells with a nearly unlimited proliferative capacity, as shown by serial transplantation experiments in which grafted hepatocytes could repopulate the injured liver parenchyma of host mice for at least 12 consecutive transplantations.^4^ The liver is the only terminally differentiated adult tissue with extensive regenerative capacity.

In mice, proliferating hepatocytes undergo rapid transcriptional reprogramming, which results in the reactivation of developmental gene expression programs.^5^ A variety of factors reside at the core of the transcriptional regenerative program, including E2Fs, ETS, CEBPB, MYC, and JUN.^5,6^ These transducers are activated through priming factors and mitogenic upstream signals, most notably growth factors and Wnts.^7^

Our current understanding of the mechanisms underlying liver regeneration is almost exclusively derived from rodent models. This is compounded by the fact that 2D cultures of primary human hepatocytes (PHH), which are conventionally used to identify factors that promote cell cycle re-entry, are short-lived and characterized by cell cycle arrest.^8^ Methods to induce sustained expansion of PHH in a variety of culture methodologies have been reported recently.^9–12^ In these studies, a fraction of hepatocytes entered the cell cycle through the use of complex media formulations that contained redundant signaling cues. While these studies have opened the door for attractive therapeutic strategies, they have not disentangled the regulatory architecture that controls cell cycle re-entry in quiescent hepatocytes.

Utilizing an organotypic 3D model of human liver cells, we report growth factor-free conditions that trigger cell cycle re-entry of PHH. Specifically, we identified that the activation of NFκB and the inhibition of TGFβ signaling are necessary and sufficient for human hepatocyte priming, while Wnt/β-catenin signaling constitutes the critical mitogenic pathway. In contrast, murine hepatocyte proliferation requires growth factors. Furthermore, using chemogenomic screening coupled with high-content assays of cell health and secondary pharmacology, we identified novel hepatocyte-specific regulators of proliferation, including the critical roles of polycomb repressive complex 2 (PRC2), as well as of bromodomain families I, II, and IV.

## METHODS

### Cell culture

Organotype culture of cryopreserved primary human liver cells was conducted as described.^13^ The demographics and available medical history of the donors are shown in Supplemental Table 1, http://links.lww.com/HEP/I52. The suppliers QPS, BioIVT, and KaLy-Cell collected informed consent from each donor or the subject’s legally authorized representative in accordance with the HHS regulations for the protection of human subjects (45 CFR §46.116 and §46.117) and Good Clinical Practice (ICH E6). All experiments were conducted using hepatocyte monocultures unless where clearly stated that spheroids were generated as co-cultures of hepatocytes with primary human KCs at a 1:4 stoichiometry.

Cryopreserved primary murine hepatocytes from CD-1 mice were commercially obtained (BioIVT, USA) and cultured under identical conditions. The recombinant proteins and small molecules used for spheroid exposure are described in Supplemental Table 2, http://links.lww.com/HEP/I53. For pharmacological inhibition of NF-κB signaling, treatment with BAY-11-7082 was started 24 hours before the cytokine-based proliferation-inducing cocktail. The study was approved by the Ethics Committee of Karolinska Institutet, Stockholm, Sweden under permit number protocol ID08995-2020.

### Liver repopulation experiments

For repopulation experiments, we used non-obese diabetic mice, in which *Fah*, *Rag2*, and *Il2rg* were knocked out (“FRGN mice”).^14,15^ We used 5–6-week-old mice, originally obtained from Yecuris Corporation (Tualatin) and maintained on a PicoLab High Energy Mouse Diet with 18.9% protein (LabDiet through International Product Supplies Ltd.) supplemented with 2-(2-nitro-4-trifluoromethylbenzoyl)-1,3-cyclohexanedione. Mice were injected with ~1 million PHH per mouse either as suspensions or spheroids cultured in 96-well Elplasia plates (Corning) of PHH at the Karolinska Institute Animal Facility under the approved ethical protocol ID08995-2020. After transplantation, the mice were cycled on and off 2-(2-nitro-4-trifluoromethylbenzoyl)-1,3-cyclohexanedione to support the proliferation of human FAH-proficient hepatocytes. Serological human albumin was monitored biweekly using the Quantitative Human Albumin ELISA Quantitation Kit (Bethyl Laboratory).

### Gene expression analyses

RNA was isolated using a Qiazol lysis reagent (QIAGEN). The expression of target genes was evaluated using Taqman probes (Supplemental Table 3, http://links.lww.com/HEP/I54) in a 7500 Fast Real-Time PCR system (Applied Biosystems). Gene expression was quantified using the *ΔΔCt* method. For RNA sequencing, samples were processed using the New England Biolabs Next Ultra II Directional RNA Library Prep Kit (New England Biolabs) and sequenced on a NovaSeq6000 instrument (Illumina) at GenomeScan BV (Leiden, Netherlands). Raw data were processed using the RTA3.4.4 pipeline and Bcl2fastq (v2.20) conversion software (Illumina). Genes with an average number of transcripts per million mapped reads > 1 across all samples were analyzed using Qlucore (Lund, Sweden). Significantly enriched pathways were identified based on the KEGG Pathway Database using the WebGestalt toolbox.^16^ For motif activity analyses, RNA-Seq data were obtained from previously published data on dedifferentiating and redifferentiating hepatocytes.^17^ Transcription factor activity patterns were inferred using the ISMARA algorithms.^18^

### Immunofluorescence and EdU staining

Spheroids were fixed in 4% paraformaldehyde for 1–2 h, preserved overnight in a 30% sucrose solution, embedded in OCT (Sakura), and sectioned into 10 µm sections using a cryostat NX70 (Thermo Fisher). For immunofluorescence, sections were blocked and permeabilized using a 5% bovine serum albumin (Sigma Aldrich) in 0.25% Triton solution for 2 h and incubated with primary antibody overnight at 4°C (Supplemental Table 4, http://links.lww.com/HEP/I55). For labeling S-phase, 10 µM EdU (Sigma Aldrich/Merck) was added into the culture medium and revealed by incubation for 30 minutes in staining solution (0.1 M Tris pH 7.5, 2 mM CuSO_4_, 5 µM Alexa 647-azide (Thermo Fisher), 100 mM ascorbic acid). Nuclei were counterstained using DAPI (Thermo Fisher). Nuclear p21 intensity quantifications were conducted using FIJI software by measuring EdU and p21 channel intensities using a binarized mask based on the DAPI channel.

### Knock-down experiments

siRNA constructs against *RELA* (s11915) and *CTNNB1* (s438) were purchased from Thermo Fisher. The siRNAs were resuspended in RNase-free water as 20 µM stocks and transfected into cells at a final exposure concentration of 50 nM using lipofectamine RNAiMAX (Invitrogen).

### Phosphoproteomics

Frozen spheroid samples were heated for 10 min at 95°C in a lysis buffer containing 4% sodium dodecyl sulfate and sonicated using a probe sonicator. Proteomic sample preparation consisted of reduction and alkylation, methanol chloroform precipitation, and digestion of the proteins by lysyl endopeptidase (LysC, Wako) overnight and trypsin for 6 h. Peptides of the individual samples were labeled with TMTpro 16plex, pooled, and desalted using SepPack (Waters). The peptide mixture was fractionated by high-pH off-line fractionation and concatenated into 8 fractions for phosphopeptide enrichment, which was performed using MagReSyn TiO2 magnetic beads (ReSyn Biosciences). Enriched phosphopeptide samples were analyzed by liquid chromatography (LC) tandem mass spectrometry (MS) on an Orbitrap Fusion Lumos Tribrid mass spectrometer (Thermo Fisher Scientific). A database search of the proteomics data was performed using FragPipe. All statistical comparisons were performed using two-tailed homoscedastic Student *t*-tests. The mass spectrometry proteomics data files were deposited in the Proteomics Identification Database (PXD036100). Further details are provided in the Supplemental Methods, http://links.lww.com/HEP/I56.

### Chemogenomic screening

The effect of 108 chemical probes on the proliferation of PHH in 3D spheroids was evaluated using EdU incorporation. The screening panel was enriched in epigenetic and inflammatory modifiers, including methyltransferases, acetyltransferases, and bromodomains (BRDs). For BRDs, we followed the subfamily categorization established in Re.^19^. Briefly, PHH spheroids were exposed for 5 days after spheroid formation to a medium containing 5 µM A-8301 and 3 µM CHIR99021 plus 1 µM of the respective chemical probe. Imaging of whole-mount spheroids was performed in culture plates using an Opera Phenix High-Content Screening System (PerkinElmer). Detailed descriptions of counter-screens for cytotoxicity and the evaluation of compound effects on growth rates are provided in the Supplemental Methods, http://links.lww.com/HEP/I56.

## RESULTS

### Wnt/β-catenin and growth factor signaling induce cell cycle re-entry only in permissive, dedifferentiated hepatocytes

**FIGURE 1:**
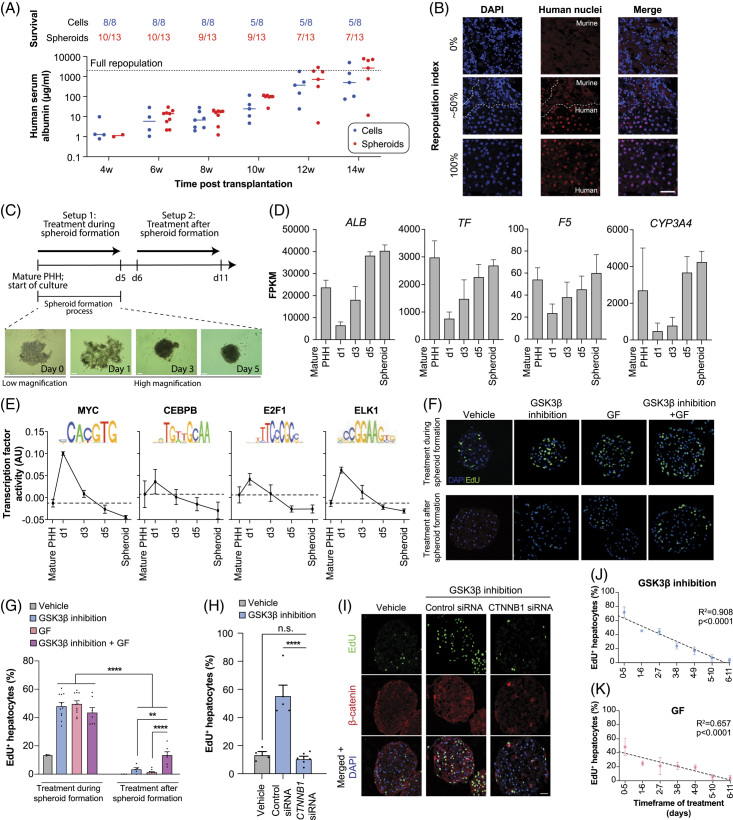
In the absence of priming factors, differentiated primary human hepatocytes are resistant to Wnt/β-catenin-mediated and growth factor-mediated cell cycle re-entry. A, Repopulation of FRGN mice with suspensions of isolated fully mature hepatocytes (“Cells”) or isogenic liver spheroids (“Spheroids”). The extent of repopulation is determined by measuring human albumin in the serum of the recipient mice every 2 weeks. Note that not every mouse was measured at each timepoint. B, Immunofluorescent images of liver sections from repopulated mice stained with antibody against human nuclei (red). Sections are counterstained with DAPI (blue), which binds to the genomic DNA of both mice and human nuclei. C, Schematic showing the different experimental setups. PHH is exposed to “GF” or the Wnt/β-catenin agonist CHIR99021 (“GSK3β inhibition”) either during the first 5 days of culture (d0–d5; setup 1) in which spheroids are forming or for 5 days (d6–d11; setup 2) after spheroid formation. D, Expression of hepatocyte markers shown as FPKM during PHH spheroid formation. E, Activity profiles of the cellular reprogramming factors MYC, CEBPB, E2F1, and ELK1 during spheroid aggregation. Logos of the binding motif sequences are shown. F, EdU stainings of PHH treated during or after spheroid formation with the Wnt/β-catenin agonist CHIR99021, the GFs EGF and HGF, or both. G, Fraction of hepatocytes re-entering the cell cycle (EdU^+^) in both experimental setups. H, Knock-down of β-catenin (*CTNNB1*) abolishes the ability of GSK3β inhibition to induce PHH proliferation. I, Representative EdU and β-catenin stainings for control and *CTNNB1* siRNA-treated PHH. J–K, Linear regression of EdU incorporation rates of PHH treated 5 days with CHIR99021 (J) or GFs (K) with progressing spheroid aggregation. Error bars indicate SEM. *, ** and **** corresponds to *p* < 0.05, *p* < 0.01 and *p* < 0.0001 compared to vehicle controls, respectively. Scale bars = 40 μm. Abbreviations: FPKM, fragments per kilobase of transcript per million mapped reads; GF, growth factors; PHH, Primary human hepatocytes.

To analyze the molecular underpinnings of human hepatocyte proliferation, we used 3D liver spheroids, which allow PHH to maintain their transcriptomic, proteomic, and metabolomic signatures for multiple weeks *in vitro*.^13,20,21^ Furthermore, we here show that human spheroids efficiently repopulate the livers of recipient FRGN mice within 14 weeks post-transplant (Figure 1A-B). These results indicate that PHH spheroids maintain essential hepatic functions and the ability to rescue a failing liver *in vivo*, which supports the translatability of our *in vitro* spheroid results.

We previously revealed that PHH transiently dedifferentiated during spheroid formation and that activation of Wnt/β-catenin signaling during this process was sufficient to evoke robust cell cycle re-entry.^17^ Here, we analyzed whether susceptibility to mitogen-induced proliferation differed as a function of hepatocellular differentiation. To this end, we compared the response of transiently dedifferentiated cells during spheroid formation (setup 1) with redifferentiated cells after spheroids were formed (setup 2; Figure 1C-E).

In agreement with previous results by us^17^ and others,^22^ we observed high fractions of cell cycle re-entry (47%) when dedifferentiated PHH were treated for 5 days with the GSK3β inhibitor CHIR99021, a well-characterized activator of Wnt/β-catenin signaling in hepatocytes (Figure 1F-G). Similar results were obtained by exposing the cells to the growth factors HGF and EGF (henceforth referred to as “GF”). To ascertain whether the observed effects were indeed due to GSK3β’s effect on Wnt signaling, we exposed cells to recombinant Wnt3a, either alone or in combination with the Wnt signaling potentiator, RSPO1. The recombinant proteins strongly induced Wnt/β-catenin activity in reporter cell lines and were able to strongly induce cell cycle entry (Supplemental Figure 1, http://links.lww.com/HEP/I57).

As GSK3β is a pleiotropic kinase with over 100 known targets,^23^ we set out to further test whether β-catenin would be its major downstream target for mediating the observed pro-proliferative effect. Importantly, upon knock-down of *CTNNB1*, the gene encoding β-catenin, we find that the pro-proliferative effect of GSK3β inhibition is significantly reduced back to baseline levels (∼11% of cells; *p* < 0.001 compared to control transfected cells, Figure 1H-I). We conclude that GSK3β inhibition by CHIR99021 mimics ligand-mediated activation in the analyzed context, and thus, we used CHIR99021 as an activator of Wnt/β-catenin signaling for all further experiments.

While Wnt/β-catenin activation strongly induced cell cycle re-entry of dedifferentiated hepatocytes, these effects were completely abolished in differentiated cells. Similarly, only dedifferentiated but not redifferentiated PHH re-entered the cell cycle upon exposure to HGF and EGF, which is surprising since these growth factors have been regarded as full mitogens.^24^ When both mitogens were combined, there was no significant increase in proliferation upon exposure during spheroid formation, whereas a significant increase from 3% to 13% (*p* < 0.001) was observed after spheroid formation (Figure 1G). During aggregation, PHH became increasingly less responsive to Wnt/β-catenin and GF-induced cell cycle re-entry over time, which paralleled cellular redifferentiation (Figure 1J-K). These data indicate that both Wnt/β-catenin and GF signaling alone are sufficient to drive the proliferation of primed PHH, whereas their capacity to trigger cell cycle re-entry decreases with increasing differentiation, and virtually no proliferation is observed in highly redifferentiated human hepatocytes. Notably, the activation of Wnt/β-catenin or GF signaling significantly increased the nuclear localization of the senescence marker p21 (Supplemental Figure 2A-B, http://links.lww.com/HEP/I58), suggesting that proliferative signaling cues induce the expression of cell cycle arrest proteins, which serve as a regulatory mechanism to limit the regenerative response, as has been reported for extended hepatectomy *in vivo*.^25^

### Cytokines synergize with Wnt/β-catenin to induce cell cycle re-entry of quiescent PHH

**FIGURE 2:**
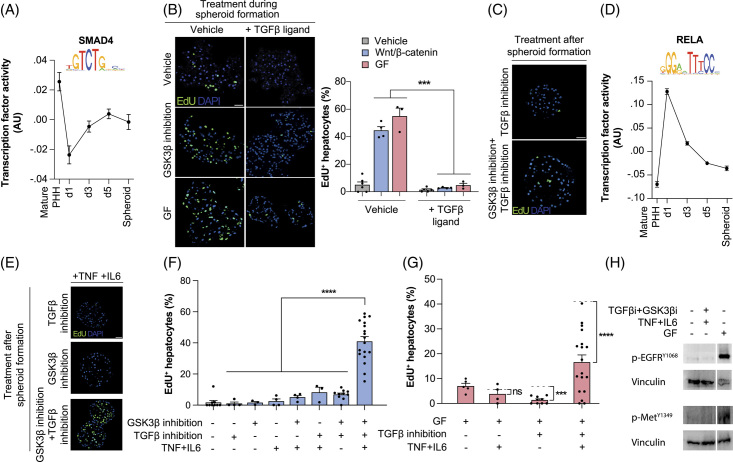
Activation of cytokines and inhibition of TGFβ signaling renders primary human hepatocytes susceptible to Wnt/β-catenin-mediated proliferation. A, The activity profile of the TGFβ signal transducer SMAD4 is decreased during spheroid aggregation. The logo depicts the analyzed SMAD4 binding motif. B, Activation of TGFβ signaling using recombinant ligand blocks proliferation of hepatocytes during spheroid aggregation induced by GSK3β inhibition or growth factors. C, EdU staining shows that in fully formed spheroids, TGFβ inhibition is not sufficient to render cells susceptible to the Wnt/β-catenin-mediated induction of proliferation. D, Activity of the NFκB transducer RELA (p65) during spheroid formation. The logo depicts the analyzed RELA binding motif. E–F, EdU staining (E) and quantification thereof (F) for simultaneous repression of TGFβ and exposure to a cocktail of pro-inflammatory cytokines (IL6 and TNF) and GSK3β inhibitor (CHIR99021). G, While GF activates proliferation in TGFβ inhibited hepatocytes treated with cytokines, the synergistic effect is significantly lower than in spheroids treated with GSK3β inhibitor (indicated by dashed lines for each condition). H, Western blots for EGFR^Y1068^ and MET^Y1349^ using phospho-specific antibodies show that co-treatment with TGFβ/GSK3β inhibition and cytokines induces hepatocyte proliferation without affecting growth factor receptor phosphorylation. Error bars indicate SEM. **, *** and **** corresponds to *p* < 0.01, *p* < 0.001 and *p* < 0.0001, respectively. Abbreviations: GF, growth factors

To investigate the molecular mechanisms involved in human hepatocyte priming, we first focused on TGFβ signaling, as genetic ablation of TGFβ receptors in mice results in accelerated liver regeneration after partial hepatectomy.^26^ TGFβ signaling was reduced in primed PHH, (Figure 2A) and the activation of TGFβ signaling during spheroid aggregation completely abolished Wnt/β-catenin-mediated or GFs-mediated PHH cell cycle re-entry (Figure 2B). However, the inhibition of TGFβ was not sufficient to induce proliferation of quiescent differentiated PHH, either alone or in combination with Wnt/β-catenin activation (Figure 2C).

Next, we analyzed the effects of acute-phase pro-inflammatory cytokines on hepatocyte proliferation, given that NF-κB signaling was markedly increased during spheroid aggregation (Figure 2D). However, neither IL6 nor TNFα induced PHH cell cycle entry alone or in combination with either Wnt/β-catenin activation or TGFβ inhibition in differentiated spheroids (Figure 2E-F). However, when cytokines, TGF-β inhibition, and Wnt/β-catenin activation were combined, extensive proliferation was observed. This finding is striking as it constitutes to our knowledge, the first GF-free condition to induce proliferation of mature fully differentiated PHH. Notably, while GF and Wnt/β-catenin activation resulted in identical stimulation of hepatocyte proliferation during spheroid aggregation (compare Figure 1D-E), GFs synergized considerably less with cytokine signaling and TGFβ inhibition than Wnt/β-catenin activation (Figure 2G). Proliferation was not only independent of extrinsically added GFs but also of GF signaling at the receptor level, as evidenced by lack of EGFR^Y1068^ and MET^Y1349^ phosphorylation in spheroids treated with TGFβ inhibitor, GSK3β inhibitor, IL6, and TNFα (Figure 2H and Supplemental Figure 3, http://links.lww.com/HEP/I59).

To test the effects of inflammatory signaling in a more physiological context, we generated co-cultured spheroids of PHH with CD163^+^/Lyz^+^ primary KCs. In line with *in vivo* findings during liver regeneration in rodents,^7,27^ human KCs produced TNF and IL1β, as well as GFs and Wnts, and significantly increased proliferation of PHH in the mixed spheroids (*p* < 0.05; Supplemental Figure 4, http://links.lww.com/HEP/I60). We thus conclude that human KCs produce molecular cues, such as cytokines, GFs, and Wnt ligands, which can participate in cell priming and activation of human hepatocyte proliferation.

### Stimulation of hepatocyte regeneration is specific to NFκB-stimulating cytokines

**FIGURE 3:**
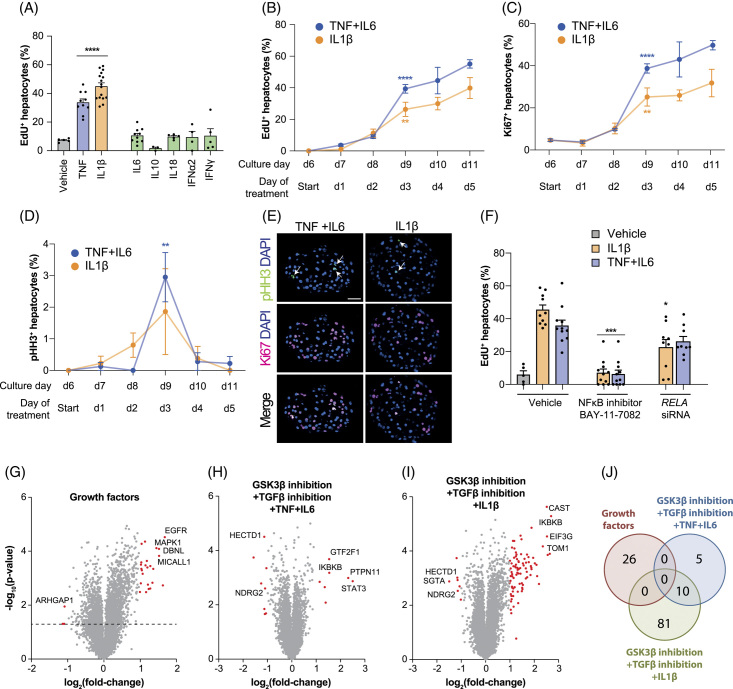
Stimulation of hepatocyte regeneration requires canonical NFκB signaling. (A) Fraction of PHH re-entering cell cycle under different NFκB-activating (TNF and IL1β) and other cytokines or interferons. (B–D) Temporal dynamics of cell cycle re-entry and progression in PHH spheroids as shown by EdU incorporation (S-phase) (B), Ki67 expression (cell cycle marker) (C), and phosphorylation of histone H3 (pHH3; mitosis marker) (D). (E) Representative images for Ki67 and pHH3 immunostainings at day 3 of treatment. (F) Inhibition of NFκB signaling using the small molecule inhibitor BAY-11-7082 or siRNA-mediated *RELA* knock-down reduces cell cycle re-entry. All treatments in panels A–F are combined with TGFβ inhibition and GSK3β inhibition. (G–I) Volcano plots of phosphoproteomic data show changes in phosphorylation status in spheroids treated with GF (G), GSK3β inhibitor, TGFβ inhibitor, IL6, and TNF (H) or GSK3β inhibitor, TGFβ inhibitor, and IL1β (I). Differentially phosphorylated proteins are shown in red (FC > 2; *p* < 0.05). (J) Venn diagram showing that the differentially phosphorylated proteins do not overlap between GF and GF-free conditions, indicating that the elicited intracellular signal transduction cascades are fundamentally different. Error bars indicate SEM. *, **, ***, and **** correspond to *p* < 0.05, *p* < 0.01, *p* < 0.001, and *p* < 0.0001 compared to the respective vehicle or control siRNA, respectively. Scale bars = 40 μm. Abbreviations: GF, growth factors; GO, gene ontology; pHH3, phosphorylation of histone H.

To evaluate the cytokine-specificity of cell cycle stimulation, we compared the effects of the major pro-inflammatory and anti-inflammatory cytokines (Figure 3A). In contrast to the pro-inflammatory cytokines IL1β and TNFα, no significant effects were observed with IL6, which is not an NF-κB activator, or with anti-inflammatory cytokines or interferons. These findings suggest that in human hepatocytes, only specific pro-inflammatory cytokines exert pro-proliferative effects and that the key signaling axes converge in the NFκB pathway.

The highest increase in PHH cell cycle entry and S-phase was observed between days 2 and 3 of treatment and only marginally increased thereafter (Figure 3B-C). EdU incorporation data was corroborated by staining for phosphorylated histone H3, an established marker of active mitosis (Figure 3D-E). As histone H3 is only phosphorylated from early prophase until late anaphase,^28^ its window is much shorter than that of other proliferation markers, such as EdU or Ki67, and the fraction of positive cells at a given timepoint is thus expected to be considerably lower.

Importantly, treatment with the pharmacological NF-κB inhibitor BAY-11-7082 abrogated the proliferative response, confirming the key role of pro-inflammatory cytokine signaling in hepatic regeneration (Figure 3F). Similarly, knock-down of *RELA* resulted in reduced phosphorylation of its encoded NFκB-signaling transducer p65 and decreased PHH cell cycle re-entry (Figure 3F, Supplemental Figure 3C, http://links.lww.com/HEP/I59). This is consistent with *in vivo* studies that reported remarkably impaired liver regeneration in knockout mice for TNF receptors and IL6, which led to significantly increased mortality rates after partial hepatectomy.^29,30^

To further compare the intracellular signal transduction network in spheroids stimulated with pro-inflammatory cytokines to that in spheroids exposed to GFs, we conducted phosphoproteomic analyses. Overall, we identified 17,914 phosphopeptides distributed across 4576 proteins. In GF-treated spheroids, GF signaling transducers, such as MAPK1, and proteins involved in GF receptor trafficking (MICALL1) were among the most significant hits (Figure 3G; Supplemental Table 5, http://links.lww.com/HEP/I61). In contrast, we observed significant phosphorylation of the NF-κB regulator IKBKB and the Wnt signaling inhibitor NDRG2 in treatments that included cytokines (Figure 3H-I). Importantly, the overall phosphorylation signatures differed drastically between conditions, further demonstrating that GF-free proliferation conditions elicit fundamentally different intracellular signal transduction networks (Figure 3J).

### RNA sequencing identifies hepatocyte dedifferentiation as a critical step in induction of proliferation

**FIGURE 4:**
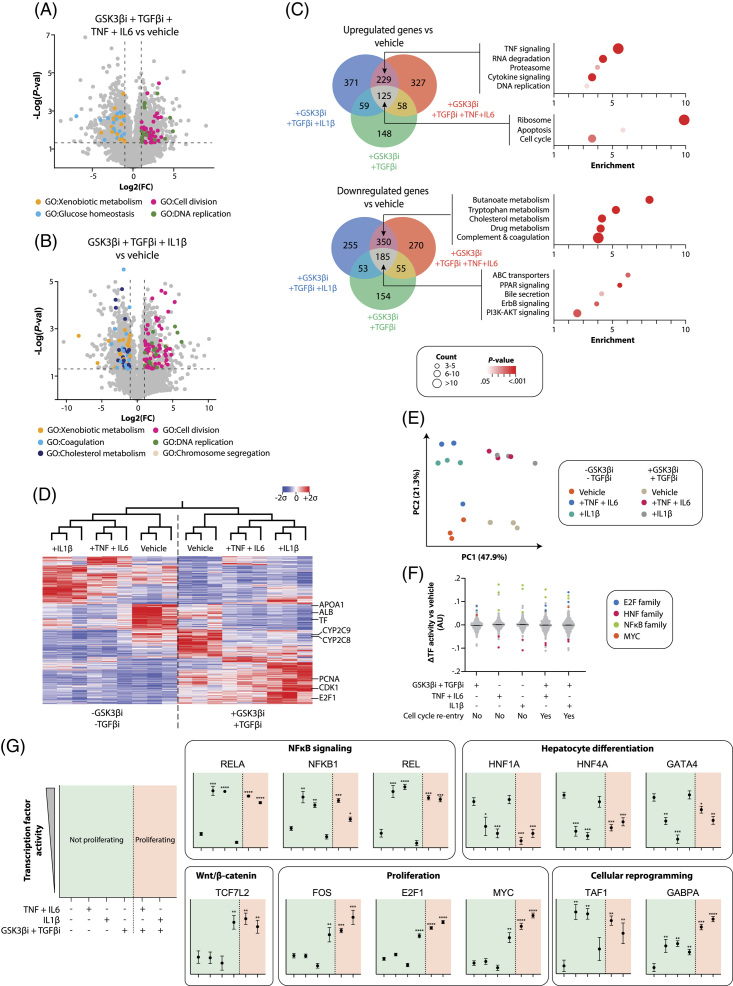
RNA sequencing identifies NFκB signaling and repression of hepatic maturation factors as key steps for hepatocyte cell cycle re-entry. (A–B) Volcano plots showing upregulated and downregulated genes compared to vehicle. Genes associated with different GO terms are colored. Hepatocyte spheroids were treated for 48 h with GSK3β inhibitor (“GSK3βi”), TGFβ inhibitor (“TGFβi”), TNF, and IL6 (A) or with GSK3βi, TGFβi, and IL1β (B). (C) Venn diagram showing the overlap of significantly upregulated and downregulated genes (FC > 2; *p* < 0.05 in a heteroscedastic *t*-test) between TGFβi/GSK3βi and acute-phase cytokines. The associated top significantly enriched pathways are indicated. (D) Mean-centered sigma-normalized heatmap representation of differentially expressed genes after hierarchical clustering (*F*-test, q < 0.05). (E) PCA of the top 100 transcription factor motif activities inferred in triplicates from RNA sequencing data. (F) Difference in transcription factor motif activity (ΔTF) between the different treatment conditions and the vehicle in hepatocyte spheroids. (G), Transcription factor motif activity for NFκB members, key hepatocyte differentiation factors, important cell cycle regulators, and transcription factors implicated in cellular reprogramming. Abbreviations: GO, gene ontology; PCA, Principal component analysis

RNA sequencing revealed that cell cycle and DNA replication genes were upregulated in cytokine-based proliferation cocktails, while genes associated with xenobiotic metabolism, coagulation, and carbohydrate and cholesterol metabolism genes, were downregulated (Figure 4A-B). Acute-phase cytokines and TGFβ/GSK3β inhibition elicited distinct cellular responses with genes involved in the cell cycle being enriched in spheroids treated with TGFβ and GSK3β inhibitors, irrespective of co-treatment with acute-phase cytokines, whereas DNA replication was enriched only in the co-treated spheroids (Figure 4C-E). These results indicate that TGFβ and GSK3β inhibition renders cells permissive for cell cycle entry but requires NF-κB activation to enter the S-phase.

TGFβ and GSK3β inhibition alone entailed a significant induction of E2F1 as well as MYC, whereas acute-phase cytokines alone strongly induce NF-κB and repress the activity of members of the HNF family and GATA4 (Figure 4F-G). Combining both TGFβ/GSK3β inhibition and cytokine signaling resulted in the integration of the individual transcription factor profiles, as well as in further amplification of E2F and MYC activity. Combined TGFβ/GSK3β inhibition and NF-κB activation moreover repressed YAP/TAZ-mediated signaling (Supplemental Figure 5, http://links.lww.com/HEP/I62), a known regulator of liver size control^31^ and the regulation of hepatocyte maturation both *in vivo* ^32^ and *in vitro*.^33^ In addition, a remarkable increase in TAF1 and GABPA activity was observed, which are transcription factors that promote the expression of ribosomal proteins essential for cellular proliferation.

### The mitogenicity of Wnt/β-catenin signaling and GFs is species-dependent

To evaluate whether the GF-free proliferation conditions we identified were specific to human hepatocytes, we tested the response to Wnt/β-catenin activation, TGFβ inhibition, as well as GF and cytokine signaling in 3D spheroids of mature primary murine hepatocytes.

**FIGURE 5:**
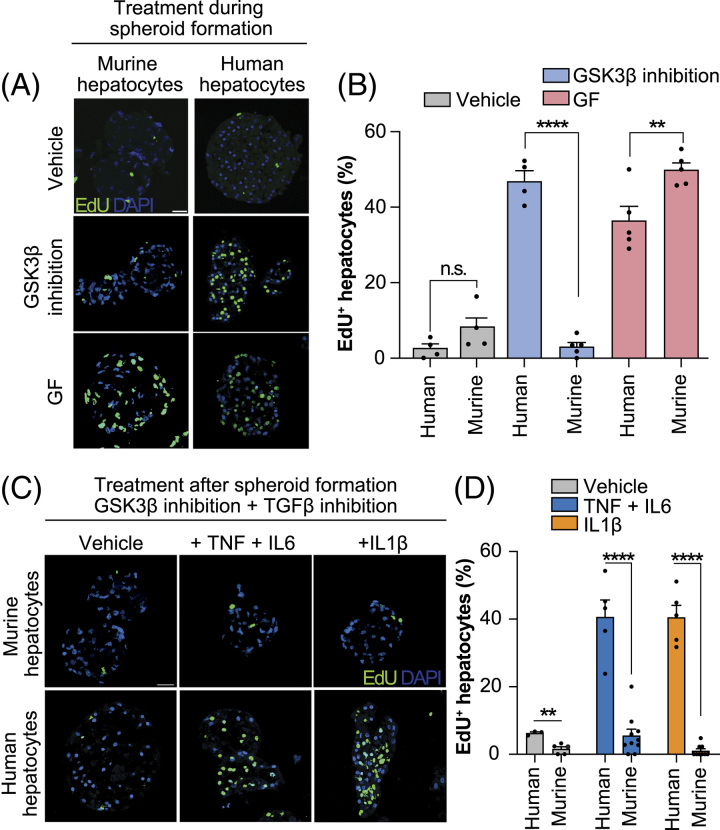
The molecular control of hepatocyte proliferation is species-specific. A–B, Representative images (A) and quantifications of EdU incorporation (B) of primary murine and human hepatocytes during spheroid formation. (C–D) Representative images (C) and quantifications of EdU incorporation (D) of formed primary human and murine hepatocytes treated with acute-phase cytokines in combination with GSK3β inhibitor and TGFβ inhibition. Error bars indicate SEM. ** and **** corresponds to *p* < 0.01 and *p* < 0.0001, respectively. Scale bars = 40 μm.

Interestingly, contrary to human hepatocytes, mouse cells did not re-enter the cell cycle under treatments based on Wnt/β-catenin activation but did so when GFs were provided (Figure 5A-B). Furthermore, murine spheroids were not susceptible to the induction of hepatocyte proliferation based on TGF-β inhibition and cytokine activation (Figure 5C-D). These data suggested that murine hepatocyte regeneration is primarily driven by GFs, as *in vivo*.^34^ In contrast, human hepatocyte proliferation is strongly dependent on Wnt/β-catenin signaling.

### Chemogenomic screen for modulators of human hepatocyte regeneration

**FIGURE 6:**
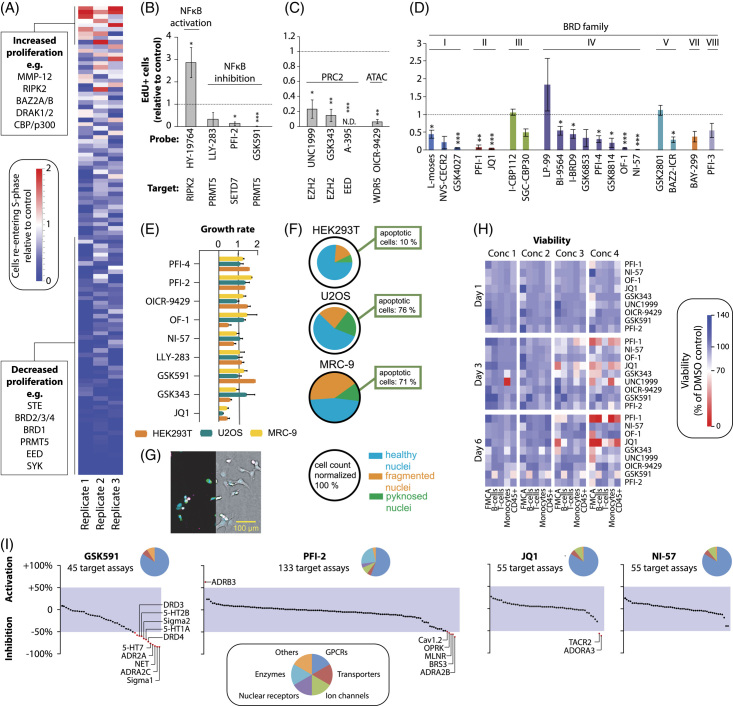
Chemogenomic screen reveals the importance of epigenetic plasticity for human hepatocyte regeneration. (A) Heatmap representation of EdU quantification data of all 108 tested probes in biological triplicates. (B) Effects of NFκB modulators on cell cycle re-entry. (C) Inhibition of the histone methyltransferase and acetyltransferase complexes PRC2 and ATAC significantly reduce proliferation. (D) Effects of BRD inhibition stratified by BRD families. N ≥ 3 for all probes in panels (B–D). (E) Analysis of the effects of selected compounds on growth rates in HEK293T, U2OS, and MRC-9 cells. Compounds were tested in duplicates, and the complete screen was performed twice. (F) Multiplex live-cell assay showing the fraction of healthy, fragmented, and pyknosed nuclei after 24 h of exposure to JQ1. The sizes of the pie charts indicate the normalized cell count. The average data of 2 biological replicates are shown. (G) Representative fluorescent (left) and brightfield image (right) of stained (blue: DNA, green: microtubule, red: mitochondria, and magenta: Annexin V apoptosis marker) U2OS cells after 24 h of JQ1 exposure. (H) Viability of a selection of hit compounds was analyzed using separate FMCA ( n = 3) analysis and flow cytometry of peripheral blood mononuclear cells for B-cells (CD19^+^), T-cells (CD3^+^), monocytes (CD14^+^), and all leukocytes (CD45^+^). Concentration ranges for each compound are provided in Supplemental Table 6, http://links.lww.com/HEP/I67. (I) Secondary pharmacology screen of selected hit compounds. In total, 150 binding assays covering G protein-coupled receptors (n = 87), ion channels (n = 12), transporters (n = 6), nuclear receptors (n = 6), enzymes (n = 31) and others (n = 8) were evaluated. Off-target effects were defined as > 50% inhibition or activation (red dots). Error bars indicate SEM. *, **, *** and **** corresponds to *p* < 0.05, *p* < 0.01, *p* < 0.001 and *p* < 0.0001, respectively. Abbreviation: ATAC, Ada2a-containing; BRD, bromodomain; FMCA, fluorometric microculture cytotoxicity assay; PRC2, polycomb repressive complex 2.

Given the substantial species differences, we used our human liver regeneration model to conduct a chemogenomic screen of 108 compounds from a diverse, highly validated library of cell-active chemical probes enriched for epigenetic regulators and modulators of immune function. In total, 25 of the 108 probes significantly altered human hepatocyte proliferation after the Benjamini-Hochberg correction (Figure 6A; Supplemental Table 6, http://links.lww.com/HEP/I63). Among the probes that increased proliferation were the BAZ2A/B inhibitor GSK2801, CBP/p300 inhibitor I-CBP112, and RIPK inhibitor HY-19764. Inhibition of RIPK kinase has previously been implicated in biasing TNF signaling away from pro-apoptotic effectors and towards NF-κB activation and cell survival^35^; however, its effects on hepatocyte proliferation have not been described. Here, we found that the inhibition of RIPK using HY-19764 significantly increased proliferation (Figure 6B). Conversely, inhibition of the methyltransferases PRMT5 and SETD7, which have been shown to activate NFκB activity,^36–38^ resulted in the ablation of hepatocyte proliferation, which further supports the pro-proliferative effect of NFκB in PHH.

We also found that epigenetic plasticity was an essential prerequisite for hepatocyte proliferation. The histone methyltransferase complex PRC2 has previously been shown to promote liver regeneration, at least in part, by inhibiting the transcription of the cell cycle inhibitors CDKN2A and CDKN2B.^39^ In agreement with these findings, we showed that specific inhibition of the PRC2 members, EED and EZH2, significantly reduced hepatocyte proliferation (Figure 6C). Similarly, we found essential roles for the histone acetyltransferase Ada2a-containing complex, as inhibition of the canonical Ada2a-containing member WDR5, which mediates the pro-proliferative effects of MYC,^40^ abolished proliferation.

Inhibition of BRD proteins of the BET subfamily (BRD family II), which block inflammatory signaling, reduced hepatocyte cell cycle re-entry, corroborating the importance of inflammatory signaling in human hepatocyte regeneration (Figure 6D). Our results indicate important roles of the BRD I family members PCAF and GCN5 as well as of multiple members of the BRD IV family, whereas no prominent effects on hepatocyte proliferation were observed for members of the BRD III, V, VII, and VIII families.

To test the cell type specificity of the observed effects, we profiled the effects of selected hit compounds on proliferation dynamics across three different cell lines, the cancer cell lines HEK293T and U2OS, and the non-transformed human fibroblast line MRC-9 (Figure 6E). No reduction in proliferation was observed with the methyltransferase inhibitors LLY-283, PFI-2, and GSK591 in any of the cell lines, whereas significant effects were observed in PHH. Similarly, only one of the 3 cell lines showed a reduction in growth rate when treated with any of the BRPF inhibitors OF-1, NI-57, or PFI-4. The BET (BRD II) inhibitor JQ1, in contrast, strongly reduced the proliferation of PHH and induced nuclear fragmentation and apoptosis, as indicated by a live-cell multiplexed assay for cell health (Figure 6F-G; Supplemental Table 7, http://links.lww.com/HEP/I64). These data indicate that the network underlying the control of proliferation only partially overlapped between PHH and cell lines typically used for compound screening.

Next, we conducted cytotoxicity analyses of selected probes by fluorometric microculture cytotoxicity assay and flow cytometry in primary human B-cells, T-cells, monocytes, and CD45^+^ leukocytes. The probes showed, if at all, only marginal cytotoxicity at the relevant concentrations, except for the BET inhibitors PFI-1 and JQ1, for which toxicity was observed at the highest concentration (Figure 6H). To further increase confidence in the specificity of the observed effects and assess biological target diversity, we conducted secondary pharmacology screening using a binding assay panel covering a total of 150 different therapeutically relevant targets (Figure 6I; Supplemental Table 8, http://links.lww.com/HEP/I65). Specifically, we focused on 2 mechanistically distinct NFκB activators, PFI-2 and GSK591, as well as the panBRPF inhibitor NI-57. GSK591 caused off-target inhibition of adrenoceptors, serotonin, dopamine, and sigma receptors. In contrast, PFI-2 and NI-57 showed no considerable off-target effects, supporting the conclusion that the observed effects on hepatocyte regeneration are likely on-target effects.

Taken together, these results indicate that the presented 3D primary human liver model constitutes a scalable platform for identifying novel factors and pathways involved in human hepatocyte regeneration through chemogenomic profiling. The obtained results confirm the importance of NFκB, uncover the importance of epigenetic regulation for proliferative control, and identify the important roles of BET and non-BET BRD proteins in human liver cell plasticity.

## DISCUSSION

The regenerative capacity of the liver relies on the highly plastic nature of hepatocytes, which allows them to switch between the quiescent and proliferative states. Murine studies have indicated that EGF and HGF are required for this switch.^34^ Additional proliferative pathways, such as Wnt/β-catenin and YAP/Hippo, have been shown to be involved in hepatocyte cell cycle re-entry; however, experiments using knockout mice have indicated that these are dispensable and not strictly required for successful liver regeneration.^41,42^ Moreover, *in vivo* models have revealed the complex crosstalk between hepatocytes and nonparenchymal cell types, which is crucial in initiating the regenerative response by providing inflammatory and mitogenic cues.^7^

While substantial mechanistic differences in liver regeneration are recognized between fish, amphibians, reptiles, and birds, the molecular control of hepatocyte regeneration in mammalian models is implicitly assumed to be highly similar.^43^ Seminal *in vitro* work on monolayer cultures of primary human and rodent hepatocytes regarded GFs as the primary mitogens, while TNF was suggested to act as a priming factor that amplified their proliferative effect.^44,45^ Similarly, while the infusion of GFs alone was sufficient to induce some DNA synthesis in uninjured rodent livers, this effect was magnified when combined with an initial priming stimulus, such as partial hepatectomy, collagenase perfusion, or portal vein cannulation.^46–48^ Here, we show that, in contrast to murine hepatocytes, Wnt/β-catenin constitutes the major mitogenic signaling axis in human liver cells. However, Wnt/β-catenin activation alone was sufficient only in dedifferentiated primed hepatocytes. Among the factors responsible for cell priming, we identified TGF-β as a central parameter. TGFβ has conventionally been considered as a factor involved in the termination of liver regeneration,^49^ and its loss is associated with the acquisition of hepatic progenitor phenotypes.^50^ Nevertheless, TGFβ signaling has more recently been proposed as a driver of hepatocyte epithelial-to-mesenchymal transition early in regeneration.^51^ Our results show that suppression of TGFβ is required for inducing the quiescent-to-proliferative switch in PHH; however, its role in PHH differentiation is uncertain. Notably, Wnt/β-catenin activation furthermore synergizes to a limited extent also with GF signaling, (Figure 1B) which could explain previous results showing that pan-frizzled activation in mice *in vivo* was sufficient to elicit cell cycle re-entry of periportal hepatocytes.^52^

In agreement with the initial priming theory,^53^ our data demonstrate a central role for the NFκB-activating cytokines in rendering hepatocytes receptive to mitogenic stimuli. In a partial hepatectomy mouse model, TNF and IL6 induce a controlled inflammatory response that amplifies hepatocyte cell cycle re-entry.^54^ In rodents, IL1β was reported to induce the expression of the EGFR ligand amphiregulin^55^; however, its prominent role in the control of hepatocyte proliferation has not been demonstrated. In this study, IL1β was as potent as TNF in cell priming. In contrast, anti-inflammatory cytokines did not increase cell proliferation in agreement with *in vivo* data.^56^ In human hepatocytes, GFs synergize with acute-phase cytokines to a lesser extent than Wnt/β-catenin to promote cell cycle re-entry.

Experiments in reductionistic *in vitro* systems offer opportunities to investigate the minimal molecular circuitry underlying proliferation and can provide new insights into the signaling networks that control human hepatocyte cell cycle re-entry. However, the observation that Wnt/β-catenin and GF signaling alone are sufficient to drive the proliferation of primed PHH, whereas their capacity to trigger cell cycle re-entry decreases with increasing differentiation, would benefit from further validations *in vivo*. Humanized mice, such as the FRGN mouse model used in this study, infused with GFs or Wnt/β-catenin agonists might provide useful tools in this regard. However, these mice exhibit an immunodeficient phenotype, which might interfere with the signaling modules identified here. Thus, careful profiling of the *in vivo* responses of differentiated and undifferentiated PHH to different signaling cues constitutes an important complement to understand human hepatocyte cell cycle control.

RNA sequencing analysis indicated that repression of hepatic differentiation and inhibition of transcription factors of the HNF family constitutes a critical step for hepatocyte cell cycle entry. This hypothesis is supported by several lines of evidence. First, GSK3β inhibition alone is sufficient to induce the proliferation of dedifferentiated PHH in the absence of cytokines (compare Figure 1E-H). Second, the loss of mature hepatic signatures is a common feature between the induction of proliferation in spheroids and organoid culture systems (Supplemental Figure 6, http://links.lww.com/HEP/I66). Lastly, similar observations have been made *in vivo* where proliferating hepatocytes have been shown to reactivate an early postnatal-like expression signature after partial hepatectomy.^5^

The mechanisms underlying the plastic nature of the liver are intriguing, as no other organ displays these characteristics. It is conceivable that these differences are based on specific epigenetic signatures. Our results pinpoint PRC2 and Ada2a-containing complexes as bromodomain members of the BET family (BRD family II) as critical epigenetic regulators of hepatocyte plasticity. Furthermore, using orthogonal probes, we showed that besides BET proteins, members of the BRD I (histone acetyltransferase) and BRD IV families (scaffolding proteins and transcriptional regulators) affect hepatocyte regeneration.^19^ These results provide the first evidence implicating non-BET BRDs in primary human cell plasticity and regeneration and incentivize further investigation of these protein families for mechanistic and translational studies.

In summary, we identified that cell cycle re-entry in human hepatocytes is driven by Wnt/β-catenin signaling in synergy with acute-phase pro-inflammatory cytokines under the permissiveness of TGFβ signaling. Importantly, GFs may be more potent mitogens for rodent hepatocytes, highlighting the relevance of using sophisticated *in vitro* culture systems based on adult primary human cells.

## Supplementary Material

## References

[1] Michalopoulos GK. Liver regeneration. J Cell Physiol. 2007;213:286–300. 17559071 10.1002/jcp.21172 PMC2701258

[2] Miyaoka Y Ebato K Kato H Arakawa S Shimizu S Miyajima A. Hypertrophy and unconventional cell division of hepatocytes underlie liver regeneration. Curr Biol. 2012;22:1166–1175. 22658593 10.1016/j.cub.2012.05.016

[3] MacDonald RA. Lifespan of liver cells: Autoradiographic study using tritiated thymidine in normal, cirrhotic, and partially hepatectomized rats. Arch Intern Med. 1961;107:335–343. 13764742 10.1001/archinte.1961.03620030023003

[4] Wang MJ Chen F Li JX Liu CC Zhang HB Xia Y. Reversal of hepatocyte senescence after continuous in vivo cell proliferation. Hepatology. 2014;60:349–361. 24711261 10.1002/hep.27094

[5] Chembazhi UV Bangru S Hernaez M Kalsotra A. Cellular plasticity balances the metabolic and proliferation dynamics of a regenerating liver. Genome Res. 2021;31:576–591. 33649154 10.1101/gr.267013.120 PMC8015853

[6] Li J Campbell JS Mitchell C McMahan RS Yu X Riehle KJ. Relationships between deficits in tissue mass and transcriptional programs after partial hepatectomy in mice. Am J Pathology. 2009;175:947–957. 10.2353/ajpath.2009.090043 PMC2731115 19700759

[7] Michalopoulos GK. Principles of liver regeneration and growth homeostasis. Compr Physiol. 2013;3:485–513. 23720294 10.1002/cphy.c120014

[8] Vinken M Decrock E Doktorova T Ramboer E De Vuyst E Vanhaecke T. Characterization of spontaneous cell death in monolayer cultures of primary hepatocytes. Arch Toxicol. 2011;85:1589–1596. 21479951 10.1007/s00204-011-0703-4

[9] Katsuda T Kawamata M Hagiwara K Takahashi R Yamamoto Y Camargo FD. Conversion of terminally committed hepatocytes to culturable bipotent progenitor cells with regenerative capacity. Cell Stem Cell. 2017;20:41–55. 27840021 10.1016/j.stem.2016.10.007

[10] Peng WC Logan CY Fish M Anbarchian T Aguisanda F Álvarez-Varela A. Inflammatory cytokine TNFα promotes the long-term expansion of primary hepatocytes in 3D culture. Cell. 2018;175:1607–1619.e15. 30500539 10.1016/j.cell.2018.11.012 PMC6497386

[11] Hu H Gehart H Artegiani B LÖpez-Iglesias C Dekkers F Basak O. Long-term expansion of functional mouse and human hepatocytes as 3D organoids. Cell. 2018;175:1591–1606.e19. 30500538 10.1016/j.cell.2018.11.013

[12] Zhang K Zhang L Liu W Ma X Cen J Sun Z. In vitro expansion of primary human hepatocytes with efficient liver repopulation capacity. Cell Stem Cell. 2018;23:806–819.e4. 30416071 10.1016/j.stem.2018.10.018

[13] Bell CC Hendriks DFG Moro SML Ellis E Walsh J Renblom A. Characterization of primary human hepatocyte spheroids as a model system for drug-induced liver injury, liver function and disease. Sci Rep. 2016;6:25187. 27143246 10.1038/srep25187 PMC4855186

[14] Wilson EM Bial J Tarlow B Bial G Jensen B Greiner DL. Extensive double humanization of both liver and hematopoiesis in FRGN mice. Stem Cell Res. 2014;13:404–412. 25310256 10.1016/j.scr.2014.08.006 PMC7275629

[15] Srinivasan RC Zabulica M Hammarstedt C Wu T Gramignoli R Kannisto K. A liver‐humanized mouse model of carbamoyl phosphate synthetase 1‐deficiency. J Inherit Metab Dis. 2019;42:1054–1063. 30843237 10.1002/jimd.12067

[16] Liao Y Wang J Jaehnig EJ Shi Z Zhang B. WebGestalt 2019: gene set analysis toolkit with revamped UIs and APIs. Nucleic Acids Res. 2019;47:W199–W205. 31114916 10.1093/nar/gkz401 PMC6602449

[17] Oliva‐Vilarnau N Vorrink SU Ingelman‐Sundberg M Lauschke VM. A 3D cell culture model identifies Wnt/β‐catenin mediated inhibition of p53 as a critical step during human hepatocyte regeneration. Adv Sci. 2020;7:2000248. 10.1002/advs.202000248 PMC7404138 32775153

[18] Balwierz PJ Pachkov M Arnold P Gruber AJ Zavolan M van Nimwegen E. ISMARA: Automated modeling of genomic signals as a democracy of regulatory motifs. Genome Res. 2014;24:869–884. 24515121 10.1101/gr.169508.113 PMC4009616

[19] Filippakopoulos P Picaud S Mangos M Keates T Lambert JP Barsyte-Lovejoy D. Histone recognition and large-scale structural analysis of the human bromodomain family. Cell. 2012;149:214–231. 22464331 10.1016/j.cell.2012.02.013 PMC3326523

[20] Bell CC Lauschke VM Vorrink SU Palmgren H Duffin R Andersson TB. Transcriptional, functional, and mechanistic comparisons of stem cell-derived hepatocytes, HepaRG Cells, and three-dimensional human hepatocyte spheroids as predictive in vitro systems for drug-induced liver injury. Drug Metab Dispos. 2017;45:419–429. 28137721 10.1124/dmd.116.074369 PMC5363699

[21] Vorrink SU Ullah S Schmidt S Nandania J Velagapudi V Beck O. Endogenous and xenobiotic metabolic stability of primary human hepatocytes in long‐term 3D spheroid cultures revealed by a combination of targeted and untargeted metabolomics. FASEB J. 2017;31:2696–2708. 28264975 10.1096/fj.201601375R PMC5434660

[22] Unzu C Planet E Brandenberg N Fusil F Cassano M Perez‐Vargas J. Pharmacological induction of a progenitor state for the efficient expansion of primary human hepatocytes. Hepatology. 2019;69:2214–2231. 30549291 10.1002/hep.30425 PMC6519263

[23] Beurel E Grieco SF Jope RS. Glycogen synthase kinase-3 (GSK3): Regulation, actions, and diseases. Pharmacol Ther. 2015;148:114–131. 25435019 10.1016/j.pharmthera.2014.11.016 PMC4340754

[24] Michalopoulos GK Bhushan B. Liver regeneration: Biological and pathological mechanisms and implications. Nat Rev Gastroenterol Hepatol. 2021;18:40–55. 32764740 10.1038/s41575-020-0342-4

[25] Lehmann K Tschuor C Rickenbacher A Jang JH Oberkofler CE Tschopp O. Liver failure after extended hepatectomy in mice is mediated by a p21-dependent barrier to liver regeneration. Gastroenterology. 2012;143:1609–1619. 22960658 10.1053/j.gastro.2012.08.043

[26] Romero-Gallo J Sozmen EG Chytil A Russell WE Whitehead R Parks WT. Inactivation of TGF-β signaling in hepatocytes results in an increased proliferative response after partial hepatectomy. Oncogene. 2005;24:3028–3041. 15735717 10.1038/sj.onc.1208475

[27] Yang J Mowry LE Nejak-Bowen KN Okabe H Diegel CR Lang RA. Beta‐catenin signaling in murine liver zonation and regeneration: A Wnt‐Wnt situation!. Hepatology. 2014;60:964–976. 24700412 10.1002/hep.27082 PMC4139486

[28] Hendzel MJ Wei Y Mancini MA Van Hooser A Ranalli T Brinkley BR. Mitosis-specific phosphorylation of histone H3 initiates primarily within pericentromeric heterochromatin during G2 and spreads in an ordered fashion coincident with mitotic chromosome condensation. Chromosoma. 1997;106:348–360. 9362543 10.1007/s004120050256

[29] Cressman DE Greenbaum LE DeAngelis RA Ciliberto G Furth EE Poli V. Liver failure and defective hepatocyte regeneration in interleukin-6-deficient mice. Science. 1996;274:1379–1383. 8910279 10.1126/science.274.5291.1379

[30] Yamada Y Kirillova I Peschon JJ Fausto N. Initiation of liver growth by tumor necrosis factor: Deficient liver regeneration in mice lacking type I tumor necrosis factor receptor. Proc National Acad Sci. 1997;94:1441–1446. 10.1073/pnas.94.4.1441 PMC19810 9037072

[31] Grijalva JL Huizenga M Mueller K Rodriguez S Brazzo J Camargo F. Dynamic alterations in Hippo signaling pathway and YAP activation during liver regeneration. Am J Physiol Gastrointest Liver Physiol. 2014;307:G196–G204. 24875096 10.1152/ajpgi.00077.2014

[32] Yimlamai D Christodoulou C Galli GG Yanger K Pepe-Mooney B Gurung B. Hippo pathway activity influences liver cell fate. Cell. 2014;157:1324–1338. 24906150 10.1016/j.cell.2014.03.060 PMC4136468

[33] Sun P Zhang G Su X Jin C Yu B Yu X. Maintenance of Primary Hepatocyte Functions In Vitro by Inhibiting Mechanical Tension-Induced YAP Activation. Cell Rep. 2019;29:3212–3222. 31801084 10.1016/j.celrep.2019.10.128

[34] Paranjpe S Bowen WC Mars WM Orr A Haynes MM DeFrances MC. Combined systemic elimination of MET and epidermal growth factor receptor signaling completely abolishes liver regeneration and leads to liver decompensation. Hepatology. 2016;64:1711–1724. 27397846 10.1002/hep.28721 PMC5074871

[35] Hayden MS Ghosh S. Regulation of NF-κB by TNF family cytokines. Semin Immunol. 2014;26:253–266. 24958609 10.1016/j.smim.2014.05.004 PMC4156877

[36] Wei H Wang B Miyagi M She Y Gopalan B Huang DB. PRMT5 dimethylates R30 of the p65 subunit to activate NF-κB. Proc National Acad Sci. 2013;110:13516–13521. 10.1073/pnas.1311784110 PMC3746871 23904475

[37] Hartley A-V Wang B Mundade R Jiang G Sun M Wei H. PRMT5-mediated methylation of YBX1 regulates NF-κB activity in colorectal cancer. Sci Rep-uk. 2020;10:15934. 10.1038/s41598-020-72942-3 PMC7522246 32985589

[38] Li Y Reddy MA Miao F Shanmugam N Yee JK Hawkins D. Role of the Histone H3 Lysine 4 Methyltransferase, SET7/9, in the Regulation of NF-κB-dependent Inflammatory Genes. J Biol Chem. 2008;283:26771–26781. 18650421 10.1074/jbc.M802800200 PMC2546554

[39] Bae WK Kang K Yu JH Yoo KH Factor VM Kaji K. The methyltransferases enhancer of zeste homolog (EZH) 1 and EZH2 control hepatocyte homeostasis and regeneration. FASEB J. 2015;29:1653–1662. 25477280 10.1096/fj.14-261537 PMC4415007

[40] Thomas LR Wang Q Grieb BC Phan J Foshage AM Sun Q. Interaction with WDR5 promotes target gene recognition and tumorigenesis by MYC. Mol Cell. 2015;58:440–452. 25818646 10.1016/j.molcel.2015.02.028 PMC4427524

[41] Sekine S Gutiérrez PJA Yu-Ang lan B Feng S Hebrok M. Liver‐specific loss of β‐catenin results in delayed hepatocyte proliferation after partial hepatectomy. Hepatology. 2007;45:361–368. 17256747 10.1002/hep.21523

[42] Lu L Finegold MJ Johnson RL. Hippo pathway coactivators Yap and Taz are required to coordinate mammalian liver regeneration. Exp Mol Medicine. 2018;50:e423. 10.1038/emm.2017.205 PMC5992983 29303509

[43] Delgado-Coello B. Liver regeneration observed across the different classes of vertebrates from an evolutionary perspective. Heliyon. 2021;7:e06449. 33748499 10.1016/j.heliyon.2021.e06449 PMC7970152

[44] Block GD Locker J Bowen WC Petersen BE Katyal S Strom SC. Population expansion, clonal growth, and specific differentiation patterns in primary cultures of hepatocytes induced by HGF/SF, EGF and TGF alpha in a chemically defined (HGM) medium. J Cell Biology. 1996;132:1133–1149. 10.1083/jcb.132.6.1133 PMC2120765 8601590

[45] Kirillova I Chaisson M Fausto N. Tumor necrosis factor induces DNA replication in hepatic cells through nuclear factor kappaB activation. Cell Growth Differ. 1999;10:819–828. 10616907

[46] Webber EM Godowski PJ Fausto N. In vivo response of hepatocytes to growth factors requires an initial priming stimulus. Hepatology. 1994;19:489–497. 8294105

[47] Liu ML Mars WM Zarnegar R Michalopoulos GK. Collagenase pretreatment and the mitogenic effects of hepatocyte growth factor and transforming growth factor‐α in adult rat liver. Hepatology. 1994;19:1521–1527. 8188184

[48] Patijn GA Lieber A Schowalter DB Schwall R Kay MA. Hepatocyte growth factor induces hepatocyte proliferation in vivo and allows for efficient retroviral‐mediated gene transfer in mice. Hepatology. 1998;28:707–716. 9731563 10.1002/hep.510280317

[49] Karkampouna S ten Dijke P Dooley S Kruithof-de Julio M. TGFβ Signaling in Liver Regeneration. Curr Pharm Design. 2012;18:4103–4113. 10.2174/138161212802430521 22630085

[50] Thenappan A Li Y Kitisin K Rashid A Shetty K Johnson L. Role of transforming growth factor β signaling and expansion of progenitor cells in regenerating liver. Hepatology. 2010;51:1373–1382. 20131405 10.1002/hep.23449 PMC3001243

[51] Oh S-H Swiderska-Syn M Jewell ML Premont RT Diehl AM. Liver regeneration requires Yap1-TGFβ-dependent epithelial-mesenchymal transition in hepatocytes. J Hepatol. 2018;69:359–367. 29758331 10.1016/j.jhep.2018.05.008 PMC6349217

[52] Hu S Liu S Bian Y Poddar M Singh S Cao C. Single-cell spatial transcriptomics reveals a dynamic control of metabolic zonation and liver regeneration by endothelial cell Wnt2 and Wnt9b. Cell Rep Med. 2022;3:100754. 36220068 10.1016/j.xcrm.2022.100754 PMC9588996

[53] Fausto N Campbell JS Riehle KJ. Liver regeneration. Hepatology. 2006;43:S45–S53. 16447274 10.1002/hep.20969

[54] Böhm F Köhler UA Speicher T Werner S. Regulation of liver regeneration by growth factors and cytokines. EMBO Mol Med. 2010;2:294–305. 20652897 10.1002/emmm.201000085 PMC3377328

[55] Berasain C García-Trevijano ER Castillo J Erroba E Lee DC Prieto J. Amphiregulin: An early trigger of liver regeneration in mice. Gastroenterology. 2005;128:424–432. 15685553 10.1053/j.gastro.2004.11.006

[56] Yin S Wang H Park O Wei W Shen J Gao B. Enhanced Liver Regeneration in IL-10–Deficient Mice after Partial Hepatectomy via Stimulating Inflammatory Response and Activating Hepatocyte STAT3. Am J Pathology. 2011;178:1614–1621. 10.1016/j.ajpath.2011.01.001 PMC3078469 21435447

