# Supplementary material for: Wnt/β-catenin and NFκB signaling synergize to trigger growth factor-free regeneration of adult primary human hepatocytes
Source: Hepatology. 2023 Oct 23;79(6):1337–51. doi: 10.1097/HEP.0000000000000648 (PMC11095891; doi:10.1097/HEP.0000000000000648)
Supplement: Supplementary file 1 [file hep-79-1337-s001.docx]

**Supplementary Table 1: Medical and demographic information of PHH and Kupffer cell (KC) donors.** PHH = primary human hepatocytes; KC = primary human Kupffer cells.

| **Donor** | **Cell type** | **Sex** | **Ethnicity** | **Age** | **Cause of death** | **Relevant medical and social history** |
| --- | --- | --- | --- | --- | --- | --- |
| 1 | PHH | F | Caucasian | 47 | N/A | N/A |
| 2 | PHH | F | Hispanic | 30 | Head trauma | Smoker, narcotic dependency |
| 3 | PHH | F | African | 27 | Anoxia | Respiratory disease, ventilator dependent quadriplegia |
| 4 | PHH | F | Caucasian | 39 | Cardiac arrest | Hypertension, narcotic dependency, smoker |
| 5 | PHH | M | Caucasian | 25 | Head trauma | Smoker, marijuana dependency |
| 6 | PHH | M | Caucasian | 8 | N/A | None documented |
| 7 | KC | F | Caucasian | 49 | Head trauma | None documented |
