# Supplementary material for: Wnt/β-catenin and NFκB signaling synergize to trigger growth factor-free regeneration of adult primary human hepatocytes
Source: Hepatology. 2023 Oct 23;79(6):1337–51. doi: 10.1097/HEP.0000000000000648 (PMC11095891; doi:10.1097/HEP.0000000000000648)
Supplement: Supplementary file 2 [file hep-79-1337-s002.docx]

**Table 2. Small molecules and recombinant peptides used in cell culture treatments.**

| **Small molecule or protein** | **Activity** | **Supplier** | **Catalog #** | **Concentration** |
| --- | --- | --- | --- | --- |
| CHIR99021 | Activates Wnt/β-catenin signaling by inhibiting GSK3β | TOCRIS | 4423 | 3 µM |
| A83-01 | Inhibition of TGF-β signaling by blocking ALK5 | Sigma Aldrich/MERCK | SML0788 | 5 µM |
| BAY-11-7082 | Inhibition of NFκB signaling by blocking IKK | Sigma Aldrich/MERCK | B5556 | 5 µM |
| C59 | Inhibition of Wnt secretion by inhibiting PORCN | Abcam | ab142216 | 10 nM |
| Human EGF | Growth factor | R&D Systems | 236-EG-200 | 10 ng/ml |
| Human HGF | Growth factor | Peprotech | 100-39H | 40 ng/ml |
| Human TGFβ1 | Proinflammatory cytokine | Peprotech | 100-21 | 10 ng/ml |
| Human IL-6 | Proinflammatory cytokine | Peprotech | 200-06 | 10-30 ng/ml |
| Human TNF | Proinflammatory cytokine | Peprotech | 300-01A | 10-30 ng/ml |
| Human IL-1β | Proinflammatory cytokine | Peprotech | 200-01B | 10-30 ng/ml |
| Human IL-10 | Anti-inflammatory cytokine | R&D Systems | 217-IL-010 | 10 ng/ml |
| Human IFN⍺2 | Proinflammatory cytokine | Biolegend | 592701 | 10 ng/ml |
| Human IFNɣ | Proinflammatory cytokine | Biolegend | 570202 | 10 ng/ml |
| Human IL-18 | Proinflammatory cytokine | Biolegend | 592102 | 10 ng/ml |
| Human Wnt3a | Wnt ligand | R&D Systems | 5036-WN-010 | 30-300 ng/ml |
| Human RSPO1 | Wnt potentiator | R&D Systems | 4645-RS-025 | 30-300 ng/ml |
| Murine EGF | Growth factor | Peprotech | 315-09 | 10 ng/ml |
| Murine HGF | Growth factor | Peprotech | 315-23 | 40 ng/ml |
| Murine IL-6 | Proinflammatory cytokine | Peprotech | 216-16 | 30 ng/ml |
| Murine TNF | Proinflammatory cytokine | Peprotech | 315-01A | 30 ng/ml |
| Murine IL-1β | Proinflammatory cytokine | Peprotech | 211-11B | 30 ng/ml |
