# Supplementary material for: Wnt/β-catenin and NFκB signaling synergize to trigger growth factor-free regeneration of adult primary human hepatocytes
Source: Hepatology. 2023 Oct 23;79(6):1337–51. doi: 10.1097/HEP.0000000000000648 (PMC11095891; doi:10.1097/HEP.0000000000000648)
Supplement: Supplementary file 3 [file hep-79-1337-s003.docx]

**Supplementary Table 3. Taqman probes for qPCR analyses.**

| **Gene** | **Probe ID** |
| --- | --- |
| TBP | Hs00427620_m1 |
| CD163 | Hs00174705_m1 |
| LYZ | Hs00426232_m1 |
| IL-1β | Hs01555410_m1 |
| IL-6 | Hs00174131_m1 |
| TNF | Hs00174128_m1 |
| AREG | Hs00174164_m1 |
| HGF | Hs00300159_m1 |
| Wnt2 | Hs00608224_m1 |
