# Supplementary material for: Wnt/β-catenin and NFκB signaling synergize to trigger growth factor-free regeneration of adult primary human hepatocytes
Source: Hepatology. 2023 Oct 23;79(6):1337–51. doi: 10.1097/HEP.0000000000000648 (PMC11095891; doi:10.1097/HEP.0000000000000648)
Supplement: Supplementary file 4 [file hep-79-1337-s004.docx]

**Supplementary Table 4. Primary antibodies used in this study for IF and WB.**

| **Target gene/antigen** | **Host species** | **Application** | **Working dilution** | **Supplier** | **Catalog #** |
| --- | --- | --- | --- | --- | --- |
| KI-67 | Mouse | IF | 1:500 | Cell Signaling | #9449 |
| pHH3 | Rabbit | IF | 1:250 | Cell Signaling | #9701 |
| p21 | Rabbit | IF | 1:100 | Abcam | Ab109520 |
| CD163 | Goat | IF | 1:250 | R&D systems | AF1607-SP |
| Human Nuclear Antigen | Mouse | IF | 1:100 | Abcam | Ab191181 |
| β-catenin | Mouse | IF | 1:500 | BD Biosciences | #610153 |
| YAP1 | Rabbit | IF | 1:250 | Cell Signalling | #14074 |
| P-Met Y1349 | Rabbit | WB | 1:1000 | Cell Signalling | #3133 |
| Met | Rabbit | WB | 1:1000 | Cell Signalling | #8198 |
| P-EGFR Y1068 | Rabbit | WB | 1:1000 | Cell Signalling | #3777 |
| EGFR | Rabbit | WB | 1:500 | Santa Cruz | Sc-03 |
| P-NFκB (p65) | Rabbit | WB | 1:1000 | Cell Signalling | #3033 |
| NFκB (p65) | Rabbit | WB | 1:1000 | Cell Signalling | #8242 |
| Vinculin | Rabbit | WB | 1:10000 | Abcam | Ab129002 |
