# Supplementary material for: Wnt/β-catenin and NFκB signaling synergize to trigger growth factor-free regeneration of adult primary human hepatocytes
Source: Hepatology. 2023 Oct 23;79(6):1337–51. doi: 10.1097/HEP.0000000000000648 (PMC11095891; doi:10.1097/HEP.0000000000000648)
Supplement: Supplementary file 5 [file hep-79-1337-s005.docx]

**Supplementary Methods**

***Proteomic sample preparation***

Frozen pelleted spheroid samples were heated in a buffer composed of 4% sodium dodecyl sulfate in 20 mM EPPS(3-[4-(2-Hydroxyethyl)piperazin-1-yl]propane-1-sulfonic acid, E1894, Sigma, St. Louis, MO, USA) adjusted to pH 8.5 at 95 ^0^C for 10 min, placed on ice and sonicated using a probe sonicator (3 s on pulse followed by 3 s off pulse, 1 min, 30% amplitude) (Branson). Protein concentration in the lysates was determined using Pierce bicinchoninic assay kit (ThermoFisher Scientific) according to manufacturer’s instructions. The lysates were reduced using 5 mM dithiothreitol for 1 h at RT and alkylated using 15 mM iodoacetamide for 1 h at RT in the dark. Proteins were precipitated using methanol chloroform precipitation. Protein pellets were air dried and resuspended into 8 M urea in 20 mM EPPS pH 8.5. Urea concentration was diluted to 4 M by adding equivalent volume of 20 mM EPPS, lysyl endopeptidase (LysC, Wako) was added at a 1:100 ratio (LysC/protein, w/w) and incubated overnight at RT. Then urea concentration was reduced to 1 M and trypsin digestion was conducted at a 1:100 ratio (trypsin/protein, w/w) for 6 h at RT. TMTpro 16plex labels were resuspended into dry ACN and were added to the protein digest at a ratio of 1:4 (peptide/label, w/w), the final concentration of ACN was adjusted to 20% and labelling was performed for 2 h at RT. The reaction was quenched by adding 0.5% of hydroxylamine for 15 min at RT and the samples were all combined. The resulting sample was dried in a SpeedVac until the ACN was evaporated, acidified by adding trifluoroacetic acid (TFA), desalted using SepPack (Waters) according to manufacturer’s instructions and dried overnight in a SpeedVac. The sample was resuspended into 20 mM NH_4_OH and high-pH off-line fractionated using a Ultimate 3000 RSLCnano System (Dionex) equipped with a XBridge Peptide BEH 25 cm column of 2.1 mm internal diameter, packed with 3.5 μm C18 beads of 300 Å pores (Waters). The fractionation resulted in 96 fractions that were concatenated into 8 fractions. Samples were then dried using SpeedVac and resuspended into 10% TFA, 1M glycolic acid in 80% ACN for phosphopeptide enrichment using MagReSyn TiO_2_ magnetic beads (ReSyn Biosciences) according to manufacturer’s instructions. The enriched phosphopetides were dried overnight in a SpeedVac, resuspended into 5% formic acid (FA), desalted using StageTip (ThermoFisher Scientific) according to manufacturer's instructions and dried in a SpeedVac.

***Mass spectrometry analysis***

The dried phosphopeptides were resuspended into 2% ACN and 0.1% FA (solvent A) and injected into an UltiMate 3000 UPLC autosampler (ThermoFisher Scientific) coupled to an Orbitrap Fusion Lumos Tribrid mass spectrometer (ThermoFisher Scientific). The peptides were loaded on a trap column (Acclaim PepMap C_18_, 100 μm × 2 cm) and separated on a 50 cm long C_18_ Easy spray column (ThermoFisher Scientific). Chromatographic separation of the peptides was archived by the following gradient: 4-26 % of solvent B (98% ACN and 0.1% FA) in 135 min, to 34 % in 15 min, to 95 % in 2 min where it was kept for 3 min, before going to 4% in 2 min and hold for 8 min, resulting in a 165 min gradient. During the entire gradient the mass spectrometer was operating in positive polarity using a data-dependent acquisition mode. All mass spectra were acquired in profile mode using the Orbitrap mass analyzer. An acquisition cycle was 2 s long and consisted of one survey mass spectrum acquired at a 120`000 mass resolution from m/z 400 to 1600, with an AGC target of 1`000`000 and a maximal injection time of 50 ms. Only peptides with a charge state between +2 and +6 were isolated for MS/MS with an isolation window of 0.7 m/z units, The MS/MS spectra were recorded at a 60`000 resolution, with 35 % NCE, an AGC target of 150`000 AGC and maximal injection time of 118 ms. The dynamic exclusion time as set to 90 s.

***Proteomic data analysis***

Raw files were converted to mzML format by MSConvert (version 3.0.21258)^1^ where peak picking of profile mass spectra was enabled with the vendor-provided algorithm (ThermoFisher Scientific). The converted files were searched using the FragPipe GUI v18 using MSFragger ^2^ as search engine against the human Swissprot database (20`409 entries) with Trypsin as the digestion enzyme allowing for up to two missed cleavages. The peptide length was set to 7 – 50 amino acids and the peptide mass range to 200 – 5000 Da. Acetylation of the N-terminus, oxidation on methionine and phosphorylation on Serine, Threonine, Tyrosine (maximal occurrence was set to three) were set as variable modifications. Carbamidomethylation of cysteine residues, TMTpro on the N-terminus or lysine as fixed modifications. The precursor tolerance was set to +/-20 ppm and fragment mass tolerance to 20 ppm. Peptide-spectrum matches (PSMs) were adjusted to a 1% false discovery rate with Percolator ^3^ as part of the Philosopher toolkit (v4.4)^4^. Isobaric quantification was performed with TMT-Integrator ^5^ using the default settings for phosphoproteomics experiments. All further data processing was performed in R (version 4.2.1). Median and variance scaled normalized multi-site results from TMT-Integrator were filtered for phosphopeptides with a localization score of at least 0.75 and no missing values across the samples. All statistical comparisons were performed based on two-tailed Student's t-test with equal variances; the differences between comparisons were reported as log2-scaled fold change values.

***Toxicity assays***

Peripheral blood mononuclear cells (PBMC) were isolated by density centrifugation and seeded at 1 million cells/ml in 48-well plates in RPMI medium supplemented with 10% calf serum, 2mM L-glutamine, 100 µg/ml penicillin, and 100 U/ml streptomycin. The probes were added at four concentrations between 1 nM and 10µM as indicated. For flow cytometry, cells were harvested, washed, and stained with CD14-FITC (clone M5E2, BD Biosciences), CD3-PerCP (UCHT1), CD19-PE-Cy7 (HIB19), CD45-PB (RPA-T8, BioLegend), and near-IR dead cell marker (Invitrogen). Analyses were performed using a Beckman Coulter Gallios instrument and FlowJo software (Tree Star Inc.). All viability data were normalized to the viability in the vehicle control (0.1% DMSO). Toxicity was measured using the Fluorometric Microculture Cytotoxicity Assay (FMCA) in three independent experiments as previously reported ^7^. Cells were seeded in 384-well plates using a Biomek 4000 pipetting robot (Beckman Coulter) and cultured overnight before the test compounds were added to triplicate wells at four concentrations for each drug. Four columns of untreated cells served as controls in each plate, whereas one column with only medium served as the blank. The survival index was calculated as the fluorescence in test wells divided by the fluorescence in control wells minus blank well readings.

***Effects of compounds on growth rates***

HEK293T (ATCC CRL-1573), U2OS (ATCC HTB-96), and MRC-9 (ATCC CCL-2) cells were seeded at a density of 2,000 cells per well in 384-well plates in DMEM (HEK293T and U2OS) or EMEM (MRC-9) supplemented with L-glutamine, 10% FBS (Gibco), and penicillin/streptomycin (Gibco). Confluency was monitored at 10x magnification in an IncuCyte (Sartorius) instrument using phase-contrast imaging before treatment and after 6h, 12h, 18h and 24h after compound exposure. Growth rates were calculated by normalizing to untreated cells and cells treated with DMSO 0.1% as reported previously ^8^. For JQ1, cells were also stained with 60 nM Hoechst33342 (Thermo Scientific), 75 nM Mitotracker red (Invitrogen), 0.3 µl/well Annexin V Alexa Fluor-680 conjugate (Invitrogen), and 25 nl/well BioTracker 488 Green Microtubule Cytoskeleton Dye (EMD Millipore). Fluorescence and cellular morphology were measured before treatment and after 12h and 24h of compound exposure using a CQ1 high-content confocal microscope (Yokogawa). Images were analyzed using CellPathfinder software (Yokogawa). Cells were detected as described ^9^ and gated using a machine learning algorithm ^10^. Data were normalized against the average number of vehicle (0.1% DMSO)-treated cells. Compounds were tested in duplicate within one experimental run, and a complete screen was performed twice.

***Secondary pharmacology screening***

Screening for off-target effects for four selected hit compounds covering a total of 150 targets was conducted by Eurofins-Cerep, using radiometric binding assays. Further evaluation of receptor binding profiles was conducted by the National Institute of Mental Health’s Psychoactive Drug Screening Program (NIMH PDSP), with reference to the PDSP online resource at https://pdsp.unc.edu/ims/investigator/web for experimental details regarding the individual assays.

***TOPFlash reporter gene assay***

HEK293A cells (300,000 cells/ml) were transfected in suspension using linear PEI (Alfa Aesar, molecular weight 25,000, PEI:DNA ratio 3:1) with 250 ng of M50 Super 8x TOPFlash (Addgene plasmid #12456), 50 ng of Renilla luciferase control plasmid (pRL-TK, Promega), and 700 ng of empty vector per ml cell suspension. Subsequently, cells were seeded into poly D-lysine-coated, white 96-well plates and washed with HBSS (HyClone) and 80µL of serum-free DMEM containing 10nM porcupine inhibitor C59 (Abcam). Next, recombinant Wnt3a and R-Spondin 1 (RSPO1; R&D Systems) were added, as indicated. 24 hours after stimulation, the cells were analyzed using a Dual-Luciferase Assay Kit (Promega), following the manufacturer’s instructions, in a Spark multimode microplate reader (Tecan).

**References**

1. Chambers, M. C. *et al.* A cross-platform toolkit for mass spectrometry and proteomics. *Nat Biotechnol* 30, 918–920 (2012).

2. Kong, A. T., Leprevost, F. V., Avtonomov, D. M., Mellacheruvu, D. & Nesvizhskii, A. I. MSFragger: ultrafast and comprehensive peptide identification in mass spectrometry–based proteomics. *Nat Methods* 14, 513–520 (2017).

3. Käll, L., Canterbury, J. D., Weston, J., Noble, W. S. & MacCoss, M. J. Semi-supervised learning for peptide identification from shotgun proteomics datasets. *Nat Methods* 4, 923–925 (2007).

4. Leprevost, F. da V. *et al.* Philosopher: a versatile toolkit for shotgun proteomics data analysis. *Nat Methods* 17, 869–870 (2020).

5. Djomehri, S. I. *et al.* Quantitative proteomic landscape of metaplastic breast carcinoma pathological subtypes and their relationship to triple-negative tumors. *Nat Commun* 11, 1723 (2020).

6. Perez-Riverol, Y. *et al.* The PRIDE database and related tools and resources in 2019: improving support for quantification data. *Nucleic Acids Res* 47, gky1106 (2018).

7. Lindhagen E, Nygren P, Larsson R. The fluorometric microculture cytotoxicity assay. *Nat Protoc* 3, 1364–1369 (2008).

8. Hafner M, Niepel M, Chung M, et al. Growth rate inhibition metrics correct for confounders in measuring sensitivity to cancer drugs. *Nat Methods* 13, 521–527 (2016).

9. Howarth A, Schröder M, Montenegro RC, et al. HighVia—A Flexible Live-Cell High-Content Screening Pipeline to Assess Cellular Toxicity. *SLAS Discov* 25, 801–811 (2020).

10. Tjaden A, Chaikuad A, Kowarz E, et al. Image based annotation of Chemogenomic Libraries for Phenotypic Screening. *Molecules* 27, 1439 (2022).
