# Supplementary figures and images for: Wnt/β-catenin and NFκB signaling synergize to trigger growth factor-free regeneration of adult primary human hepatocytes
Source: Hepatology. 2023 Oct 23;79(6):1337–51. doi: 10.1097/HEP.0000000000000648 (PMC11095891; doi:10.1097/HEP.0000000000000648)

# Supplementary Figure 2

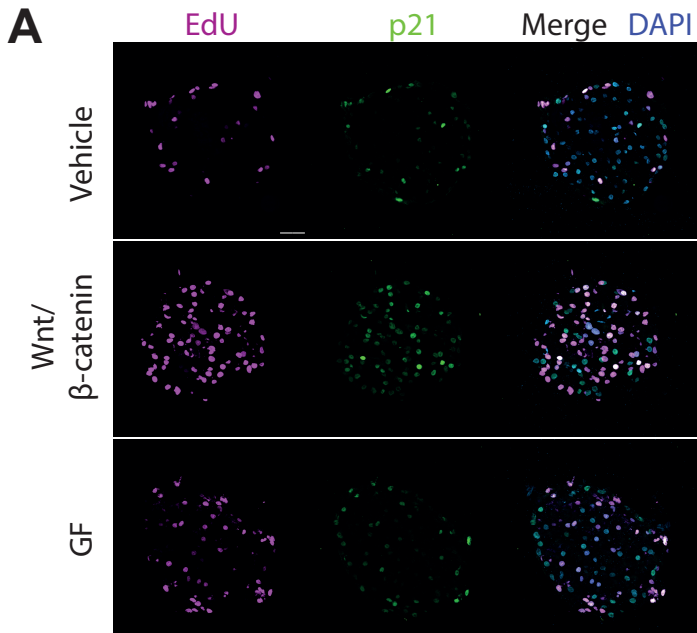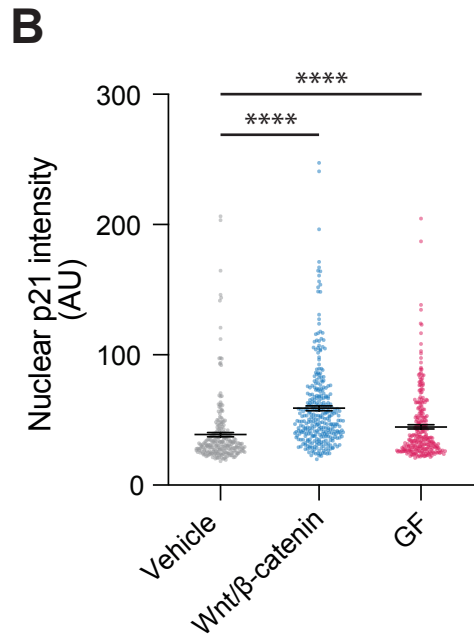

Supplement: Supplementary file 7 [file hep-79-1337-s007.pdf]

# Supplementary Figure 4

**A**

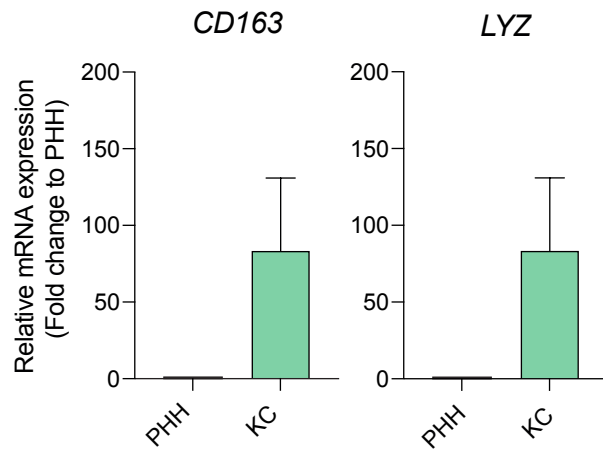

**B**

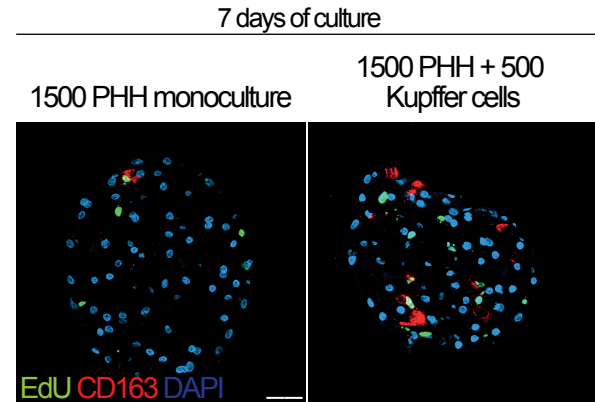

**C**

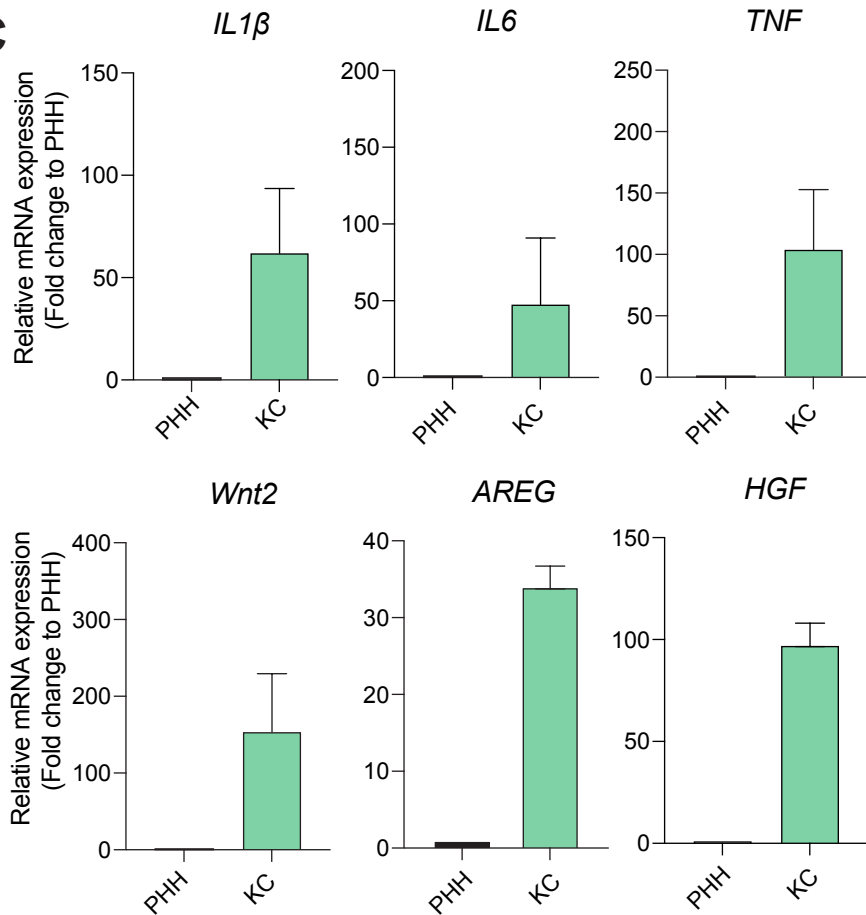

**D**

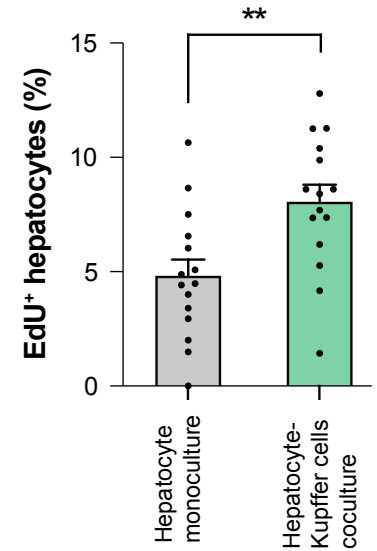

Supplement: Supplementary file 9 [file hep-79-1337-s009.pdf]

# Supplementary Figure 6

**A**

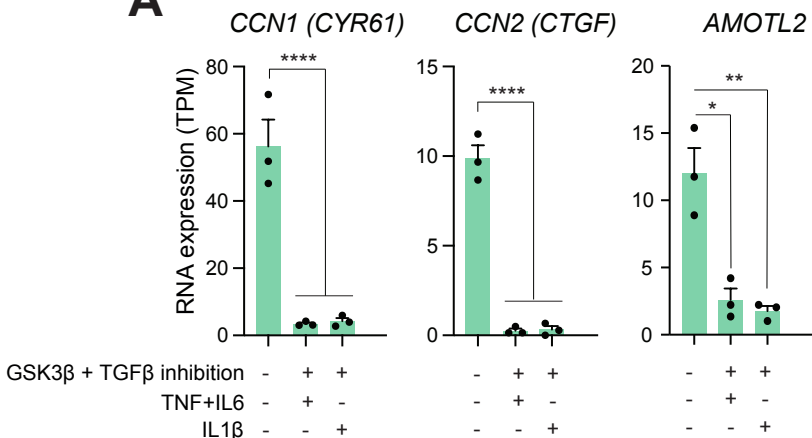

**B**

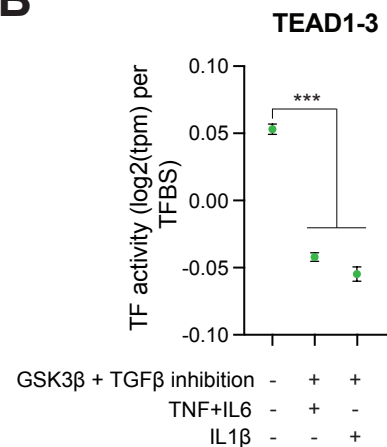

**C**

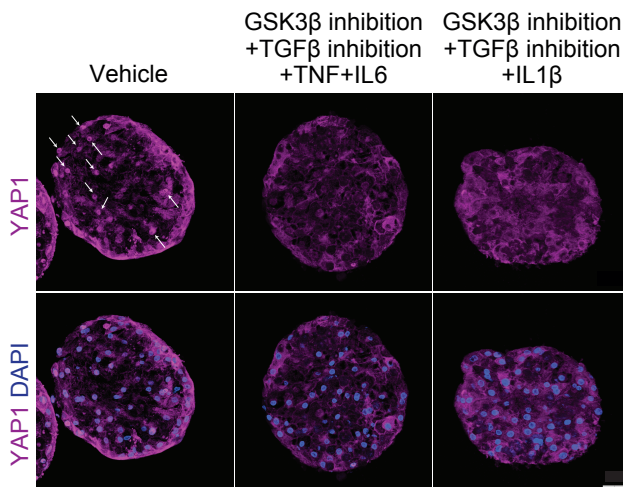

**D**

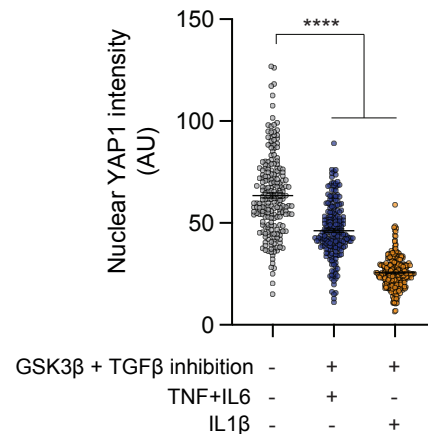

Supplement: Supplementary file 11 [file hep-79-1337-s011.pdf]
